# Calcium current homeostasis and synaptic deficits in hippocampal neurons from Kelch-like 1 knockout mice

**DOI:** 10.3389/fncel.2014.00444

**Published:** 2015-01-07

**Authors:** Paula P. Perissinotti, Elizabeth A. Ethington, Erik Almazan, Elizabeth Martínez-Hernández, Jennifer Kalil, Michael D. Koob, Erika S. Piedras-Rentería

**Affiliations:** ^1^Department of Cell and Molecular Physiology, Loyola University Chicago, Stritch School of MedicineMaywood, IL, USA; ^2^Department of Laboratory Medicine and Pathology, Institute for Translational Neuroscience, University of MinnesotaMinneapolis, MN, USA; ^3^Neuroscience Institute, Loyola University Chicago, Stritch School of MedicineMaywood, IL, USA

**Keywords:** actin-binding proteins, Kelch-like 1 protein, T-type voltage-gated calcium channels, P/Q-type voltage-gated calcium channels, LVA, HVA, synapsin I, SCA8

## Abstract

Kelch-like 1 (KLHL1) is a neuronal actin-binding protein that modulates voltage-gated Ca_V_2.1 (P/Q-type) and Ca_V_3.2 (α_1H_ T-type) calcium channels; KLHL1 knockdown experiments (KD) cause down-regulation of both channel types and altered synaptic properties in cultured rat hippocampal neurons (Perissinotti et al., 2014). Here, we studied the effect of ablation of KLHL1 on calcium channel function and synaptic properties in cultured hippocampal neurons from KLHL1 knockout (KO) mice. Western blot data showed the P/Q-type channel α_1A_ subunit was less abundant in KO hippocampus compared to wildtype (WT); and P/Q-type calcium currents were smaller in KO neurons than WT during early days *in vitro*, although this decrease was compensated for at late stages by increases in L-type calcium current. In contrast, T-type currents did not change in culture. However, biophysical properties and western blot analysis revealed a differential contribution of T-type channel isoforms in the KO, with Ca_V_3.2 α_1H_ subunit being down-regulated and Ca_V_3.1 α_1G_ up-regulated. Synapsin I levels were also reduced in the KO hippocampus and cultured neurons displayed a concomitant reduction in synapsin I puncta and decreased miniature excitatory postsynaptic current (mEPSC) frequency. In summary, genetic ablation of the calcium channel modulator resulted in compensatory mechanisms to maintain calcium current homeostasis in hippocampal KO neurons; however, synaptic alterations resulted in a reduction of excitatory synapse number, causing an imbalance of the excitatory-inhibitory synaptic input ratio favoring inhibition.

## Introduction

The actin-binding protein KLHL1 is expressed in the cytosol, axons and dendrites of neurons and in glia in the nervous system (Prag and Adams, 2003; Jiang et al., 2007; Chen et al., 2008). He and collaborators confirmed KLHL1’s physiological role in cerebellar function using targeted deletion of KLHL1 in Purkinje neurons, which resulted in dendritic deficits, mild cerebellar atrophy, abnormal gait and progressive loss of motor coordination in mice (He et al., 2006). The KLHL1 gene locus lies near genes implicated in Spinocerebellar ataxia type 8 (SCA8; Ikeda et al., 2007), a rare and slowly progressive neurological disease characterized by the presence of trinucleotide expansions in two genes: ataxin 8 (ATXN8) and ATXN8 opposite strand (ATXN8OS). Mutations in ATXN8 and ATXN8OS genes cause disruptions in multiple cellular pathways; it has been proposed that the characteristic SCA pathogenesis does not begin until calcium signaling pathways are disrupted, and calcium blockers and stabilizers have been suggested as potential treatment in the disease (Kasumu and Bezprozvanny, 2012). The 5’region of the KLHL1 gene overlaps with the 5’ end of ATXN8OS (Koob et al., 1999; Nemes et al., 2000; Ikeda et al., 2007). The expression pattern of the KLHL1 antisense strand suggests a regulatory role of KLHL1, and trans-RNA interference assays support that ATXN8OS may function as an endogenous negative regulator of KLHL1 (Nemes et al., 2000; Lin et al., 2007; Chen et al., 2008). Therefore, understanding the physiological functions of KLHL1 will help frame our understanding of the regulatory role of its antisense.

KLHL1 functions as a modulator of neurite outgrowth and process elongation, and it is also a modulator of ion channel activity (Seng et al., 2006; Aromolaran et al., 2007, 2009, 2010; Jiang et al., 2007). KLHL1 positively modulates Ca_V_2.1 (P/Q-type, α_1A_) and 3.2 (T-type α_1H_) voltage-gated calcium channel function, but it does not affect Ca_V_3.1 (T-type α_1G_) or 3.3 (T-type α_1I_) channels, nor Ca_V_1.2, 1.3, or 2.2 (Perissinotti et al., 2014). *In vitro* studies demonstrated that KLHL1 interacts with the polymerized actin cytoskeleton and with the pore-forming subunit of the Ca_V_3.2 channel. This interaction increases its recycling *via* the recycling endosome, increasing the number of active channels at the plasma membrane thus up-regulating T-type calcium current density (Aromolaran et al., 2010). Adenoviral delivery of shRNA against KLHL1 down-regulates KLHL1, which in turn decreases expression levels and current density of Ca_V_3.2 and Ca_V_2.1 channels in cultured rat hippocampal neurons; KLHL1 also reduces synapsin I levels, miniature excitatory postsynaptic current (mEPSC) frequency and the number of excitatory and inhibitory synapses (Perissinotti et al., 2014).

To corroborate the effects of endogenous KLHL1 we assessed the status of voltage-gated calcium currents and synaptic properties in cultured hippocampal neurons from the KLHL1 KO mouse model (He et al., 2006). We found a homeostatic up-regulation of α_1C_ (Ca_V_1.2) and α_1G_ (Ca_V_3.1) concomitant to decreased expression of α_1A_ and α_1H_ subunits in the KO mice, confirming the role of KLHL1 in the positive modulation of P/Q- and T-type calcium currents. Calcium current homeostasis resulted in maintenance of total somatic calcium current levels in KO hippocampal neurons. However, the synaptic deficit could not be compensated for, resulting in decreased ratio of excitatory/inhibitory synaptic inputs onto KLHL1 KO neurons, confirming a presynaptic function of KLHL1 most likely due to its effects on synapsin I, presynaptic P/Q-type channels and T-type channels.

## Materials and methods

The animal protocols used in this study were reviewed and approved by an independent Institutional Animal Care and Use Committee (IACUC).

### Mouse model

KLHL1-knockout mice were generated in the Laboratory Medicine and Pathology from the University of Minnesota (He et al., 2006).

### Cell culture

Hippocampal cultures were obtained as described (Piedras-Rentería et al., 2004). In brief, whole hippocampi were dissected from newborn rats in Hanks’ Balanced Salts Solution (HBSS) (Sigma, St. Louis, MO) supplemented with 20% of Fetal Bovine Serum (Cellgro, Herndon, VA) (20% FBS-HBSS). Hippocampi were washed with 20% FBS-HBSS and HBSS and incubated for 10 min at 37°C with trypsin type XI (3.4 mg/ml) (Sigma, St. Louis, MO) plus DNAse type I (600 U/ml) (Calbiochem, Billerica, MA). After digestion, the tissue was washed with 20% FBS-HBSS and HBSS and mechanically dissociated in HBSS supplemented with 600 U/ml DNAse I and 12 mM MgSO_4_. Cells were plated onto matrigel-coated coverslips (12 mm, Carolina Biological Supply, NC) at a density of 25,000–35,000 and kept in a 5% CO_2_ humidified atmosphere at 37°C. The media was supplemented with thymidine -β-D-arabinofuranoside (4 μM) after the second day in culture (2 DIV, days *in vitro*).

### Biochemistry

Crude membrane fractions were extracted from WT or KLHL1 KO hippocampus following standard methods (Florio et al., 1992). Samples were extracted in lysis buffer containing 50 mM Tris·HCl pH 8.0, 150 mM NaCl, 5 mM EDTA, 0.5% sodium deoxycholate, 1% Nonidet P-40 (NP-40) and 0.1% SDS supplemented with protease inhibitor cocktail (Roche, Palo Alto, CA). Protein quantification was performed using bicinchoninic acid (BCA) colorimetric assays (Pierce, Rockford, IL). Equal protein concentrations were separated by SDS-PAGE electrophoresis (5 or 8%, at 105 V for 75 min) and transferred to nitrocellulose membranes for processing (Millipore, Billerica, MA). Membranes were blocked in Tris-buffered saline (TBS) + Tween (TBST; 0.05% Tween 20) + 5% milk at room temperature, and incubated at 4°C overnight with primary antibodies against α_1A_, α_1B_, α_1I_ (Ca_V_2.1, 2.2, 3.3; 1:500–1000; Alomone, Israel), α_1C_, α_1D_ (Ca_V_1.2, 1.3; 1:1000; NeuroMab UCDavis), α_1G_ (Ca_V_3.1; 1:1000; Millipore, CA), α_1H_ (Ca_V_3.2; 1:2,000; Sta. Cruz Biotechnology, CA), synapsin I (1:1000, Millipore, CA); GAPDH levels (1:3000; Enzo Life Science, NY) were used as internal reference to normalize for protein loading. Blot membranes were cut in three portions prior to incubation with antibodies; the upper part of the membrane (110–260 kD) was processed sequentially for ion channel α_1_ subunit detection, membranes were stripped with Restore Western Blot Stripping Buffer (Pierce, IL) before re-probing. The middle membrane was processed for synapsin I (77 and 80 kD) and the lower portion of the membrane (<55 kD) was processed for GAPDH. Membranes were washed with TBST and incubated with horseradish peroxidase (HRP)-conjugated secondary antibodies (1:10,000; Pierce, IL) at room temperature for 1 h before developing with Supersignal Femto or West Dura (Pierce, IL) before analysis with a UVP Bioimaging Epichemi^3^ system (Upland, CA). Protein levels were quantified and always normalized to levels of GAPDH.

### Immunocytochemistry

Cultured WT or KLHL1-KO hippocampal neurons were fixed at 11 DIV with 4% paraformaldehyde (Electron Microscopy Sciences, Hatfield, PA) following standard methods (Perissinotti et al., 2014). Primary antibodies were diluted in blocking solution containing 2% goat serum (Jackson ImmunoResearch Laboratories, West Grove, PA) plus 0.4% saponin in PBS and incubated overnight at 4°C (MAP2, 1:1000, EnCor Biotechnology, FL; synapsin I, 1:1000, Millipore, CA; gephyrin, 1:500, SySy, Germany; GAD67 1:500, Thermo Scientific; PSD-95, 1:1000, NeuroMab; VGlut-1, 1:1000, SySy; synapsin I, 1:1000, Millipore, CA). Samples were incubated in alexa fluor-conjugated secondary antibodies (1:2,000; Life Technologies) for 1 h at room temperature. Coverslips were mounted on slides with Citiflour (Ted Pella, Redding, CA) and stored at −20°C for subsequent detection (Multiphoton Leica TCS SP5). Data was analyzed with ImageJ freeware (NIH) (Rasband, 1997–2006). Images were thresholded using the Otsu plugin. Synaptic puncta number was measured in n sample areas = 246 μm^2^ each. The JACoP plugin was used to calculate Mander’s correlation coefficients (co-localization percentages), which provides a good quantification of signal co-localization between samples of different intensities (Otsu, 1979; Bolte and Cordelières, 2006).

### Electrophysiology

Calcium currents and spontaneous miniature postsynaptic currents (mPSCs) were recorded by whole cell patch-clamp using an Axopatch 200B amplifier (Axon instruments, Union City, CA) at room temperature. Data were acquired at 1 kHz and digitized at 20 kHz using a Digidata 1322A analog-to-digital converter. Calcium currents were recorded in an external solution containing (in mM) 5 CaCl_2_, 140 TEA-Cl, 10 HEPES and 10 glucose (pH 7.4, 300 mosmol/kgH_2_O). mPSCs were recorded in an external solution containing (in mM) 135 NaCl, 5 KCl, 2 CaCl_2_, 1 MgCl_2_, 10 HEPES, 10 glucose (pH 7.4, 300 mosmol/kgH_2_O). Pipettes pulled from borosilicate glass (Warner Instruments, Hamden, CT) had resistances of 3.5–4.5 MΩ when filled with intracellular solution containing (in mM) 108 CsMeSO_3_, 4 MgCl_2_, 10 Cs-EGTA, 9 HEPES, 5 ATP-Mg, 1 GTP-Li and 15 phosphocreatine-Tris (pH 7.4, 290 mosmol/kgH_2_O). Cell capacitance was measured from a transient current evoked by a 5 mV depolarizing step from a holding potential of −90 mV.

Cells with series resistance (*R*_s_) <20 MΩ were used; *R*_s_ was compensated on line (>80%). Data were acquired with Clampex 10 and analyzed with Clampfit 10 software (Molecular Devices). Voltage control was enhanced by increasing cell impedance using extracellular TEA and intracellular cesium to block K^+^ conductances. I-V curve properties such as its negative slope and reversal potential were monitored for appropriate voltage control. For the study of calcium current properties, we avoided recording from neurons older than 10 DIV because the possibility of space-clamp problems.

#### Current-voltage relationships (I-V curves)

Currents were elicited from a holding potential (V_H_) = −90 mV or −50 mV and depolarized for 150 ms to a test potential (V_T_) = −70 to + 60 mV, in 10 mV increments. The low voltage activated (LVA) current component contribution was determined by the subtraction method (Bean, 1985): average high voltage activated (HVA) currents obtained at V_H_ = −50 mV were subtracted from those obtained at V_H_ = −90 mV at each test potential.

#### Calcium influx

Calcium influx was determined by the current integral evoked by an action potential waveform (APW; influx normalization by capacitance was not necessary as it produced similar results as the integral values reported). The LVA component was isolated by the subtraction method. The APW consisted of a digitized hippocampal action potential with a resting potential of −70 mV, an upstroke of 116 mV/ms to a peak voltage of + 50 mV, followed by a repolarizing downstroke at −65 mV/ms to a hyperpolarizing potential of −90 mV. The repolarization from after-hyperpolarization slope was 0.78 mV/ms to resting conditions (Aromolaran et al., 2012). This protocol was applied from V_H_ of −50 or −90 mV prior to clamping the voltage at −70 mV for 10 ms before the triggering of the AP, so no changes in the driving force should be expected.

#### Current kinetics

Current activation and inactivation were assessed by stepping the neuron from V_H_ = −90 mV to V_T_ = −50 to −20 mV for 150 ms. The rate of activation was estimated from the current rise time from 10% to 90% of its maximum value (rise time 10–90%); these measurements were favored over the calculation of the time constant of activation (*τ*_on_) to minimize the contribution of the inactivation process on the activation rate value. Time constant of inactivation (τ_off_) was obtained from mono-exponential fits. The time constant of deactivation (τ_deactivation_) was measured by fitting with a mono-exponential function the decaying phase of tail currents elicited from V_H_ = −90 mV to V_T_ = −30 mV and back to V_T_ = −60 to −140 mV.

#### Steady-state analysis

Steady-state activation (SSA) was analyzed with protocols stepping from V_H_ = −90 (or −50) mV to V_T_ up to 0 mV (ΔV = 10 mV) for 12 ms followed by repolarization to −100 mV to evoke inward tail currents. Data were fitted by a single Boltzmann function of the form, *I*_max_/[1+exp^*(V_50_–V)/k*^] + *m*, where I_max_ is maximal current, V_50_ is half-voltage of activation, *k* is slope factor, and *m* is baseline. Steady-state inactivation (SSI) was determined by stepping the membrane potential to various pre-pulse voltage levels (V_pre_ = −110 to 0 mV, ΔV = 10 mV) for 1 s before depolarization to a fixed test level (−30 mV) to evoke channel opening. The resulting data were also fitted to a Boltzmann function.

#### Calcium channel pharmacology

Peak calcium current amplitude was measured at 10 mV (V_H_ = −50 mV). Test pulses were elicited every 8 s. Calcium channel blockers were applied in a sequential manner, 1 μM nifedipine (L-type currents; Sigma, St. Louis, MO), 200 nM ω-Agatoxin IVA (P/Q-type currents; Alomone Labs, Jerusalem, Israel) and 2 μM ω-Conotoxin GVIA (N-type currents; Tocris, Bristol, UK). Each blocker was applied and monitored until the currents reached a new steady-state value upon inhibition (~5 min).

#### Miniature postsynaptic currents (mPSCs)

mPSCs were recorded from neurons at 10–12 DIV. Miniature excitatory postsynaptic currents (mEPSCs) were recorded at a holding potential of −60 mV for 2 min in the presence of tetrodotoxin (TTX, 1 μM; Calbiochem, Billerica, MA) and 20 μM bicuculline (Bic, Sigma, St. Louis, MO). Miniature inhibitory postsynaptic currents (mIPSCs) were recorded at a holding potential of 0 mV for 2 min in the presence of TTX (1 μM), 10 μM NBQX (2,3-Dioxo-6-nitro-1,2,3,4-tetrahydrobenzo[*f*]quinoxaline-7-sulfonamide, TOCRIS, Bristol UK) and 10 μM APV (D(−)-2-Amino-5-phosphonopentanoic acid, Sigma, St. Louis, MO).

### Statistics

Results are presented as mean ± SEM. Statistical analysis was performed with the Sigma Plot 11 Software. Statistical significance was determined by *P* < 0.05 using Student’s *t*-test, unless otherwise noted. Analysis of Variance with *post-hoc* Duncan’s Method (ANOVA) was performed when more than 2 data sets were compared. Portions of the results reported here have been presented in abstracts at national meetings. Experimental number size (*n*) is always reported in order for WT and KO (*n* = WT, KO) in the figure legends, except for Figure 2 which is reported in the text.

**Figure 1:**
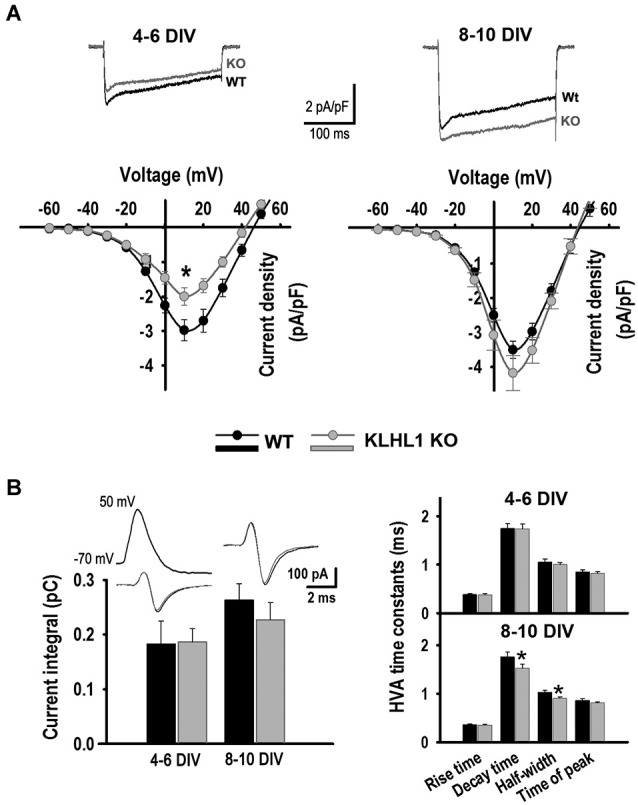
**HVA calcium currents**. **(A)** Current density I-V curves of HVA currents at 4–6 DIV and 8–10 DIV, V_H_ = −50 mV. Average traces from V_T_ = −60 to +10 mV are shown (4–6 DIV: *n* = 22, 18; 8–10 DIV: *n* = 15, 13). **(B)** Calcium current elicited by APW (*n* = 22, 34 and 21, 23); time constant values are shown to the right. *p < 0.05, Student’s *t*-test.

**Figure 2:**
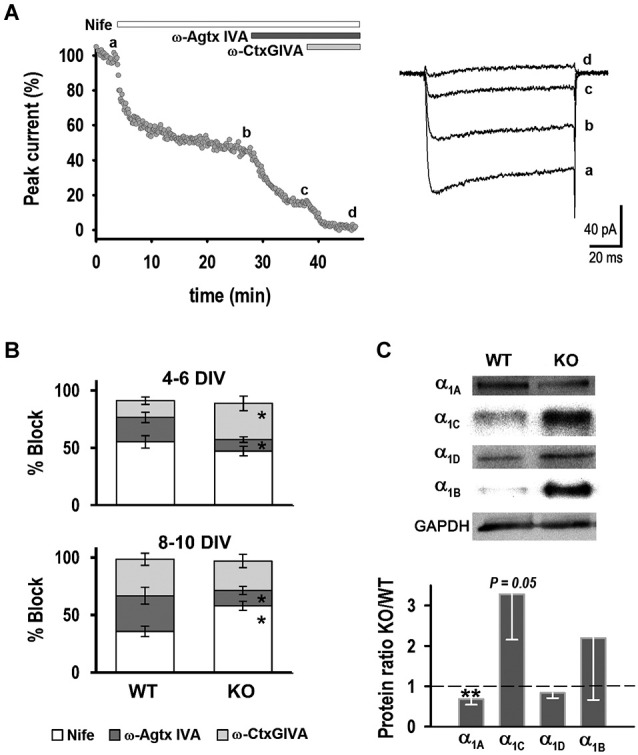
**Characterization of HVA currents**. **(A)** Example time course response of a neuron exposed to: nifedipine (1 μM), ω-Agatoxin IVA (400 nM) and ω-Conotoxin GVIA (2 μM). Right: Current traces obtained before (a) and after addition of the drugs (b–d) (V_T_ = +10 mV, V_H_ = −50 mV). **(B)** Percentage block of HVA current by antagonists in WT and KO neurons. **(C)** Western blot analysis of HVA channel subunits. Top: Examples of protein levels of HVA channel subunits in WT and KO hippocampus. Bottom: Quantification of α_1A_, α_1C_, α_1D_, α_1B_ protein ratios (KO/WT). *p < 0.05, Student’s *t*-test, **p < 0.05, paired Student’s *t*-test.

## Results

### Homeostatic regulation of voltage dependent calcium current in KLHL1 KO hippocampal neurons

We measured calcium current density from WT and KO neurons during early and late stages in culture (4–6 DIV and 8–10 DIV, respectively), using established electrophysiological protocols to assess the HVA and LVA current components (Methods).

#### HVA calcium current

The I-V curves in Figure 1A show that the HVA peak current density at 4–6 DIV was 33% smaller in KO than in WT neurons, but this difference was no longer detected at 8–10 DIV. In contrast, there were no differences in the calcium influx evoked by an APW between WT and KO at any stage in culture. However, the decay time and half-width of the APW-induced currents were smaller in KO neurons at 8–10 DIV than in WT (Figure 1B), suggesting that different HVA channels were contributing to the total HVA current during the development of KO and WT hippocampal neurons.

Sequential application of calcium channel blockers was used to identify individual HVA current components. Figure 2A shows an example where peak current was plotted *vs*. time during drug application, along with representative current traces before (a) and after application of: (b) 1 μM nifedipine; (c) 200 nM ω-agatoxin IVA; and (d) 2 μM ω-conotoxin GVIA. Figure 2B shows the fraction of current blocked by each of these antagonists at 4–6 DIV: 55 ± 5% for nifedipine (*n* = 11), 21 ± 5% for ω-agatoxin IVA (*n* = 6) and 15 ± 3% for ω-conotoxin GVIA (*n* = 5) for WT neurons. The fraction of current blocked by nifedipine was not significantly different in KO neurons (47 ± 4%, *n* = 8), whereas ω-agatoxin IVA and ω-conotoxin GVIA blocked 10 ± 3% (*n* = 6) and 32 ± 6% (*n* = 5), respectively; these fractions were significantly different from WT. In contrast, at 8–10 DIV the fraction of current blocked by nifedipine was higher in KO neurons (58 ± 4%, *n* = 16) compared to WT (36 ± 5%, *n* = 12), whereas ω-agatoxin IVA blocked a smaller fraction of current in the KLHL1-KO (13 ± 3%, *n* = 6) than in WT (31 ± 7%, *n* = 7). No differences between genotypes were observed for ω-conotoxin GVIA at 8–10 DIV (32 ± 5% and 26 ± 6% for WT and KO, *n* = 9 for each genotype). These results show that decreased levels in P/Q-type channels in KLHL1-KO neurons observed at 4–6 and 8–10 DIV were compensated for by the functional increases of N-type and L-type channels at each stage, respectively.

The expression levels of L-type α_1C_ (Ca_V_1.2) and α_1D_ (Ca_V_1.3), P/Q-type α_1A_ (Ca_V_2.1), and N-type α_1B_ (Ca_V_2.2) pore-forming subunits were determined in whole adult hippocampus by western blot (Figure 2C). KO hippocampus had lower expression of α_1A_ subunit (*n* = 6), and α_1C_ subunit levels showed a statistical tendency to higher expression compared to WT (*n* = 5, *P* = 0.05), supporting the electrophysiology data. Neither expression levels of α_1B_ (*n* = 3) subunit or α_1D_ (*n* = 4) changed in the KO.

#### LVA calcium current

**Figure 3:**
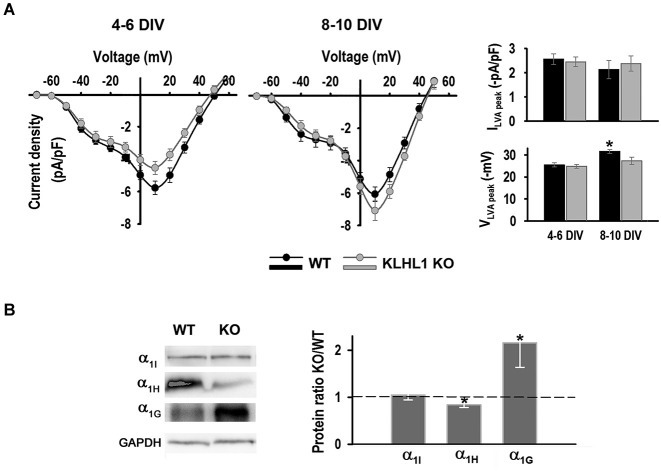
**LVA calcium currents**. **(A)** LVA and HVA current density I-V curves at 4–6 DIV and 8–10 DIV, V_H_ = −90 mV. *Right panel*: Top, LVA current density at 4–6 DIV (*n* = 22, 18) and 8–10 DIV (*n* = 15, 13). Bottom, LVA peak voltage. *p < 0.05, ANOVA. **(B)** *Left panel*: Examples of α subunit levels protein of Ca_V_3.1, 3.2 and 3.3. *Right panel*: Quantification of protein ratios (KO/WT). *p < 0.05, paired Student’s *t*-test (*n* = 7, 3, 5, from left to right).

Figure 3A shows I-V curves at early and late stages of culture. Both HVA and LVA current components can be detected in this experiment, the HVA peak component at +10 mV and the LVA peak at ~−30 mV. LVA peak current density (I_peak_) did not change during the time of culture, nor between genotypes. The LVA peak voltage (V_peak_) changed from −25 mV at 4–6 DIV to ~−32 mV at 8–10 DIV in WT neurons, but it remained unchanged in the KO. Thus, this ~7 mV left shift in the peak voltage suggests that the reported developmental change in expression of LVA channel isoforms occurred in WT but not in KO neurons (McRory et al., 2001).

Western blot analysis of whole hippocampus confirmed that the KO exhibits down-regulation of the Ca_V_3.2 α_1H_ isoform and a concomitant homeostatic increment in Ca_V_3.1 α_1G_ expression (Figure 3B); Ca_V_3.3 α_1I_ protein levels remained constant.

**Figure 4:**
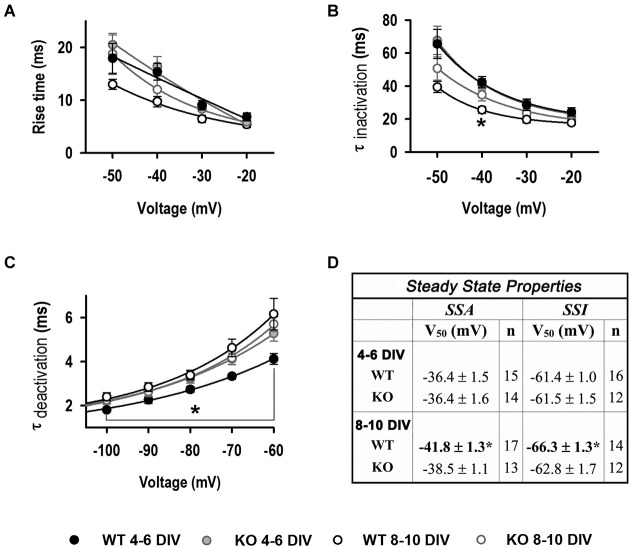
**Biophysical properties of LVA currents. (A)** Rise time (10–90%) values as function of voltage. **(B)** τ_inactivation_ as function of voltage. 4–6 DIV (*n* = 19, 22); 8–10 DIV (*n* = 10, 10) for **A**, **B**. **(C)** τ_deactivation_ as function of voltage (4–6 DIV: *n* = 14, 19; 8–10 DIV: *n* = 20, 13). ^*^Significantly different from other conditions, *p* < 0.05, ANOVA. **(D)** Steady-state inactivation (SSI) and Steady-state activation (SSA) V_50_ values. ^*^Significantly different from WT and KO at 4–6 DIV, *p* < 0.05, ANOVA.

LVA current kinetics and steady-state properties were also analyzed to assess the T-type isoform expression changes (Cain and Snutch, 2010; Figure 4). Current activation (rise time 10–90%) did not change between genotypes or during the time of culture (Figure 4A). τ_off_ was similar between genotypes and stage in culture, except for the value at −40 mV for WT at 8–10 DIV (Figure 4B). In contrast, τ_deactivation_ values were significantly slower in KO neurons compared to WT at 4–6 DIV (5.3 ± 0.3 *vs*. 4.1 ± 0.2 ms at −60 mV), but not at later stages of culture where both genotypes displayed slower τ_deactivation_, similar to those seen in KO neurons at early stages (6.1 ± 0.7 for WT and 5.7 ± 0.3 for KO) (Figure 4C). Steady-state activation (SSA) and inactivation (SSI) V_50_ properties did not change between genotypes at 4–6 DIV; however WT V_50_ values significantly shifted to negative potentials at later stages of culture (Figure 4D).

**Figure 5:**
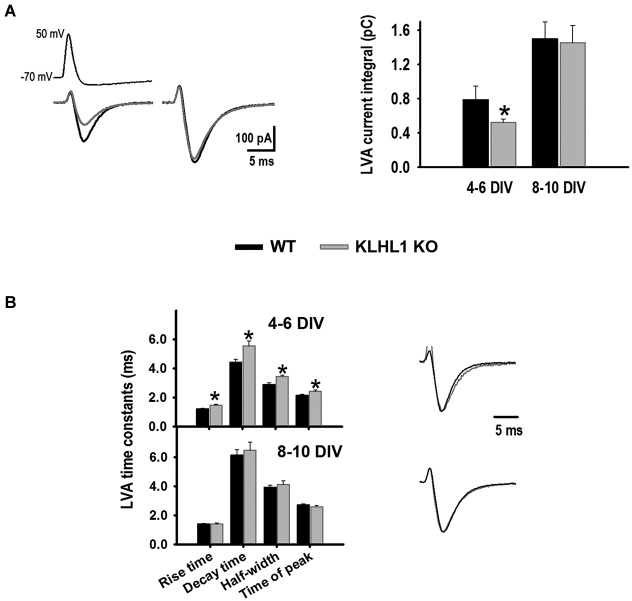
**LVA calcium currents elicited by action potential waveforms (APWs). (A)** Average trace examples during early (4–6 DIV) (left) and late (8–10 DIV) (right) stages of culture for WT (black line) and KO (gray line); average current integral values are shown to the right (*n* = 22, 34). **(B)** LVA current time constants (left) and normalized traces (right). *p < 0.05, Student’s *t*-test.

Calcium influx was assessed using APWs (Figure 5). Calcium influx *via* LVA channels was 34% smaller at 4–6 DIV in the KO compared to WT neurons, but this difference was no longer detected at 8–10 DIV. Furthermore, whereas the time constants of APW-induced LVA currents (rise time, decay time, half-width and time to peak) were slower in KO neurons at 4–6 DIV compared to WT, no differences in these constants were observed at 8–10 DIV.

### Altered synapse number in KLHL1 KO hippocampal neurons

shRNA-induced KLHL1 KD in rat hippocampal neurons results in reduction of the number of synaptic puncta, synapsin I protein levels and decreased mPSC frequency (Perissinotti et al., 2014); thus we tested the impact of the genetic ablation of KLHL1 on these synaptic properties in mice neurons.

**Figure 6:**
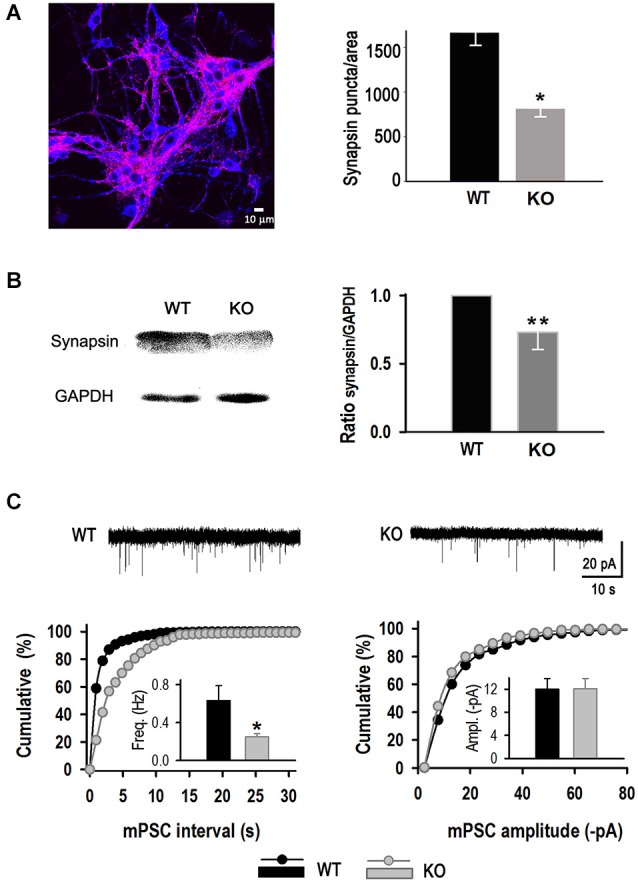
**Reduced synapse number in KLHL1 KO neurons. (A)** Quantification of synaptic puncta. *Right*, merged image of hippocampal neurons stained for synapsin I (red) and MAP2 (blue) (11 DIV). *Left*, Number of synaptic puncta per area (*n* = 11, 12). **(B)** Synapsin I quantification by Western Blot. *Left*, example blot. *Right*, data summary (*n* = 5, 5). **(C)** Miniature postsynaptic current (mPSC) analysis. *Top*, miniature PSCs example traces. *Bottom left*, mPSC frequency distribution. *Inset*, mean frequency values (Hz). *Bottom right*, mPSC amplitude distribution. *Inset*, mean amplitude value for each population (pA) (*n* = 10, 9). *p < 0.05, Student’s *t*-test; **p < 0.05, paired Student’s *t*-test.

Analysis of the number of synaptic puncta per area was performed at 11 DIV. KLHL1 KO cultures exhibited significantly fewer puncta compared to WT (*P* < 0.05). The example shown in Figure 6A depicts neurons stained for synapsin I (red) merged with the neuronal marker MAP2 (blue). Synaptic contacts decreased from 1661.2 ± 493.1 puncta per frame in WT to 809.2 ± 94.6 in KO neurons. In a separate line of experiments, quantification of synapsin I by western blot showed that protein levels were ~25% lower in the adult KO hippocampus (0.73 ± 0.126) (Figure 6B). In line with these results, mPSC properties in the absence of blockers showed lower frequency in KO neurons (0.25 ± 0.03 Hz) compared to WT (0.63 ± 0.16) (Figure 6C), confirming a decrease of synapse number in the KO. mPSC amplitude was not altered (WT: −12.0 ± 1.9 pA, KO: −12.1 ± 1.8 pA).

**Figure 7:**
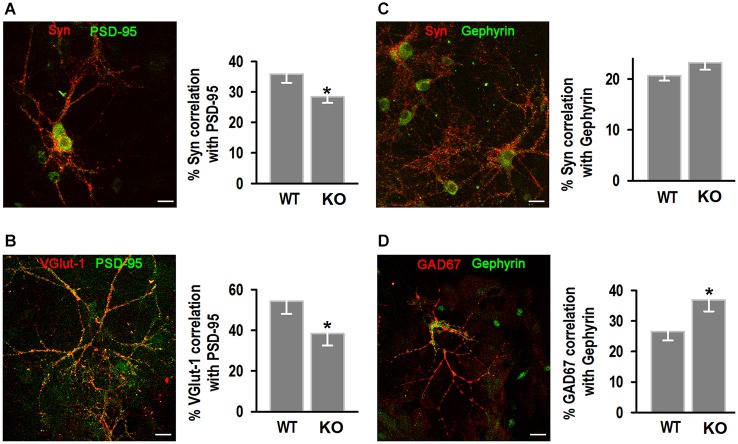
**Analysis of excitatory and inhibitory hippocampal synapses. (A,B)** Examples of excitatory synapses, identified by synapsin I (Syn) and PSD-95 antibodies **(A)** and VGlut-1 and PSD95 antibodies **(B)**; left, quantification of the % co-localization of Syn I with PSD95 (*n* = 20, 20) or VGlut-1 with PSD95 (*n* = 15, 14). **(C,D)** Inhibitory synapses, identified by Syn I and gephyrin **(C)** and GAD67 and gephyrin **(D)** antibodies; left, quantification of the % co-localization of Syn I with gephyrin (*n* = 27, 29) or GAD67 with gephyrin (*n* = 9, 11) for WT and KO. *p < 0.05, Student’s *t*-test. Scale bars = 20 μm.

To identify if the decrease in synapse number was type-specific we characterized excitatory synapses by co-localization of synapsin I (Syn I; Figure 7A), or the presynaptic glutamate vesicle transporter 1 VGlut-1 (Figure 7B) with the postsynaptic scaffolding protein PSD-95 (Woods and Bryant, 1993; Gomperts, 1996). Mander’s correlation coefficient analysis showed KO neurons exhibited less co-localization between pre- and post-synaptic excitatory markers. WT neurons exhibited 35.9 ± 2.9% co-localization of Syn I with PSD95 compared to 28.4 ± 2.0% in the KO; and 54.3 ± 6.3% co-localization of VGlut-1 with PSD-95 in WT compared to 38.3 ± 5.8% in the KO. Inhibitory synapses were identified by the presence of synapsin I (Figure 7C) or the presynaptic enzyme glutamic acid decarboxylase (GAD67, Figure 7D) and the postsynaptic scaffolding protein gephyrin (Köhler and Chan-Palay, 1983; Danglot et al., 2003). There were no differences in colocalization of Syn I with gephyrin, however signal co-localization between GAD67 and gephyrin was significantly higher in the KO (36.9 ± 3.8%) compared to WT (26.6 ± 3.0%).

**Figure 8:**
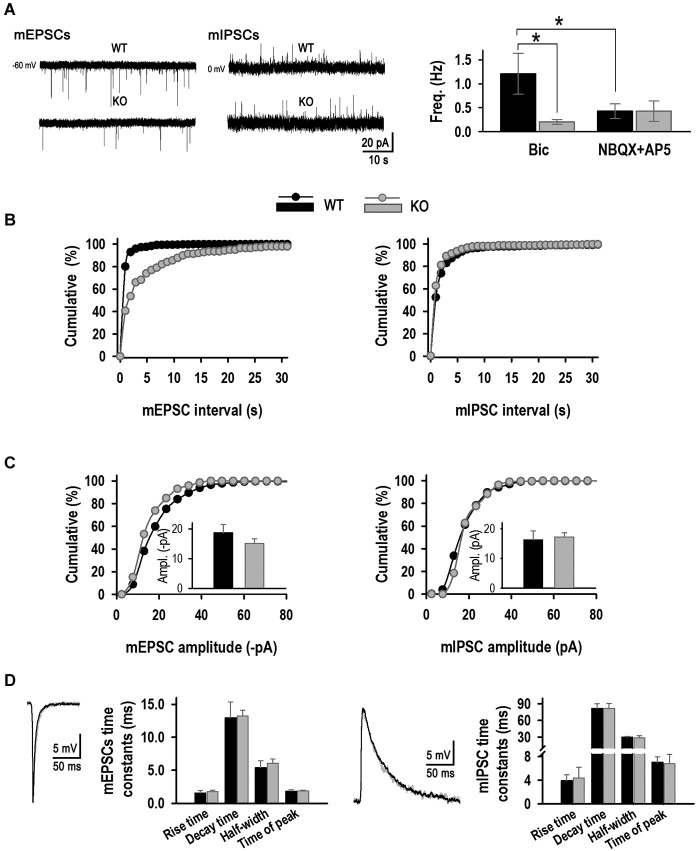
**Excitatory and inhibitory mPSC analysis. (A)** Examples of mEPSCs and mIPSC from WT and KO neurons. *Right*, Mean frequency values (Hz, *n* = 6, 7, 7, 7). **(B)** mEPSC and mIPSC frequency distribution for WT and KO. **(C)** mEPSC and mIPSC amplitude distribution for WT and KO neurons. *Inset*, mean amplitude value for each population (pA) (*n* = 6, 7, 7, 7). **(D)** Kinetic analysis. Averaged mEPSC and mIPSC traces for WT and KO synapses. *p < 0.05, ANOVA.

Excitatory and inhibitory activities were then studied in isolation using specific inhibitors (Figures 8A–D). Excitatory signals recorded in the presence of 20 μM Bic were in accordance with the co-localization experiments; excitatory activity was much lower in the KO (17% of WT), with a mEPSC frequency of 0.20 ± 0.05 Hz (*n* = 7) compared to 1.21 ± 0.43 Hz in the WT, confirming a decrease in excitatory synapse number (Figures 8A,B). Again, no changes in mEPSC amplitude were detected (−18.7 ± 2.8 pA, WT *vs*. −15.1 ± 1.5 pA, KO, Figure 8C). mIPSC activity was studied in presence of 10 μM APV and 10 μM NBQX. As expected from the Syn I-gephyrin co-localization experiments, no differences were observed in either mIPSC frequency between KO and WT genotypes (Figures 8A–C). KLHL1 KO neurons had a mean mIPSC frequency of 0.43 ± 0.21 Hz compared to 0.43 ± 0.15 Hz in the WT. mIPSC amplitude did not change: 16.3 ± 2.9 pA and 17.2 ± 1.3 pA. mIPSCs exhibited slower kinetics than mEPSCs, as expected (Shao et al., 2003; Szczot et al., 2010), but no significant changes in the kinetic properties of either excitatory or inhibitory currents were detected between groups (Figure 8D). Overall, the mEPSC/mIPSC frequency ratio was 2.8 in WT neurons, compared to 1 in the KO (Figure 8A).

## Discussion

KLHL1 function as an endogenous modulator of P/Q- and T-type calcium channels was first detected by acute KLHL1 KD experiments in rat neurons (Perissinotti et al., 2014) and is now confirmed in the KLHL1 knockout mouse model.

Hippocampal HVA currents were comprised of L-type, P/Q-type, and N type currents, as previously reported (Isaeva et al., 1998; Chameau et al., 1999; Perissinotti et al., 2014), in line with a differential contribution of HVA calcium channel subtypes at different stages *in vitro* (Chameau et al., 1999; Nudler et al., 2003). KLHL1 ablation resulted in a 33% decrease in HVA current density at early culture stages, which was compensated for by 8–10 days in culture. KO neurons exhibited decreased contribution by P/Q-type current since early stages of culture, and decreased α_1A_ levels were detected in adult hippocampus. This data is in agreement with KD experiments of KLHL1 using shRNA in cultured rat hippocampal neurons (Perissinotti et al., 2014), and also with over-expression experiments of KLHL1 *in vitro* (Aromolaran et al., 2007). N-type and L-type currents increased in KO neurons during early and late stages of culture, respectively, likely as compensatory mechanisms for the decrement in P/Q-type currents. α_1B_ subunit protein levels were similar between adult WT and KO hippocampal samples, which are representative of late stage hippocampal culture conditions, where adult neuronal phenotypes are observed. There was a possible up-regulation of the α_1C_ subunit of the L-type channel, its protein level tended to be statiscally significant by western blot analysis (*p* = 0.05); therefore increased L-type current activity may be due to post-translational mechanisms and/or increased protein levels.

LVA calcium current density was constant during the time in culture in WT neurons, as reported for cultured mouse hippocampal neurons (Chameau et al., 1999), and in contrast with acutely dissociated and cultured rat hippocampal neurons during postnatal development (Kortekaas and Wadman, 1997; Perissinotti et al., 2014). All three T-type channel isoforms (Ca_V_3.1–3.3 or α_1G-I_) are present in hippocampal neurons (McKay et al., 2006), and despite the lack of change in total current, it is known that the expression pattern of Ca_V_3.3 (α_1I_) mRNA is higher in immature compared to mature stages, whereas Ca_V_3.1 and Ca_V_3.2 expression (α_1G_ and α_1H_) predominates in mature hippocampal neurons (McRory et al., 2001). Regarding their biophysical properties, Ca_V_3.3 (α_1I_) channels have peak currents at more positive potential, display slower kinetics of activation and inactivation and have faster kinetics of deactivation compared to Ca_V_3.1 and Ca_V_3.2. α_1I_ currents also display more positive steady state V_50_ values than α_1H_/α_1G_ currents (Klöckner et al., 1999; McRory et al., 2001; Chemin et al., 2002; Perez-Reyes, 2003). Analysis of protein levels demonstrates the KO mouse model exhibited slight down-regulation of the Ca_V_3.2 α_1H_ isoform, in contrast with KD experiments where α_1H_ down-regulation was substantial (Perissinotti et al., 2014); this difference is likely due to the manifestation of compensatory mechanisms in the KO, although differences in LVA current modualtion/expression between mouse and rat models could also be involved. Ca_V_3.1 α_1G_ levels were increased and no changes in the level of Ca_V_3.3 α_1I_ expression were detected in the KO model, in line with the function of KLHL1 as a positive modulator of Ca_V_3.2 (Aromolaran et al., 2009, 2010; Perissinotti et al., 2014).

Similar to previous reports, we did not detect differences in total LVA current density between stages in culture, nor between genotypes; however analysis of biophysical properties showed V_peak_, SSA and SSI curves were shifted toward more negative values in WT neurons, with slower deactivation rates at 8–10 DIV, consistent with a decrease in the relative contribution of the α_1I_ component in WT neurons at later stages *in vitro*. In contrast, neither V_peak_, SSA nor SSI curves changed in KO neurons during their time in culture suggesting that down-regulation of α_1H_ allowed the electrophysiological detection of α_1I_ current properties, even at late stages of neuronal culture when they are normally not detectable due the preponderance of α_1G_ and α_1H_. LVA currents in KO neurons had properties similar to those seen in WT neuronal cultures at early stages *in vitro*, when α_1I_ is more abundant in hippocampal neurons. This result is in agreement with our KLHL1 knockdown study in rat hippocampal neurons (Perissinotti et al., 2014).

Ca_V_3.1 current is not modulated by KLHL1 (Aromolaran et al., 2010); and interestingly, the KO model elicited significant overexpression of α_1G_ subunit in the hippocampus. Accordingly, LVA currents in KO neurons exhibited slow time constant of deactivation from early days in culture; suggesting the presence of α_1G_ masked the contribution of the faster deactivation kinetics of α_1I_, the normally predominant component at early stages of development. The rate of deactivation was unaffected by the increase in the relative contribution of α_1G_ at later stages likely due to the concomitant decrease in α_1H_ levels, as both α_1G_ and α_1H_ have similar deactivation constants.

The unique biophysical properties of the LVA currents are more noticeable in APW experiments as previously reported (McCobb and Beam, 1991), where LVA-mediated calcium influx was 34% smaller in KO neurons at early stage, despite the similar LVA current densities detected in WT and KO. Interestingly, expression of Ca_V_3.3 in immature cultures cannot effectively provide comparable calcium influx as Ca_V_3.1 and/or Ca_V_3.2 do in mature neurons (McRory et al., 2001). The slow activation kinetics of α_1I_ channels results in small currents in response to a single action potential (Kozlov et al., 1999), uncovering the deficiency in α_1H_ subunit expression in the KO. At late stages α_1G_ and α_1H_ activate quickly and display similar current amplitudes in response to a single action potential (Kozlov et al., 1999). In agreement, we did not observe differences in the calcium influx between KO and WT genotypes at mature stages, even though these cultures displayed different α_1H_/α_1G_ expression ratios. Calcium entry through T-type channels occurs mainly during the repolarization phase of the AP, therefore the deactivation kinetics are important in tuning the current decay (Djouhri and Lawson, 1999; Chemin et al., 2002). Current deactivation and its decay time constant in response to an APW were increased in the KO neurons at early days of culture, likely due to the contribution of the slow α_1G_ component which contributes to a slowly decaying current (Chemin et al., 2002). Overall, the data show a differential contribution of T-type isoforms during development of WT and KLHL1-KO neurons *in vitro*, and a homeostatic regulation of LVA currents in the KO by increased α_1G_ levels upon down-regulation of α_1H_.

KLHL1 KD in cultured rat hippocampal neurons resulted in decreased synapsin I contents and decreased number of excitatory synapses, confirmed by decreased number of synaptic puncta and decreased mEPSC frequency. The number of inhibitory synapses was also reduced, however electrophysiological confirmation of mIPSC frequency could not be obtained in that system due to experimental difficulty (Perissinotti et al., 2014). Decreased synapsin I levels and decreased synaptic inputs onto cultured hippocampal neurons also occurred in the KLHL1 KO model. However, the reduction in the number of synapses was specific for excitatory neurons in the KO. This synaptic alteration resulted in abnormal mEPSC/mIPSC frequency ratio, favoring inhibition. In contrast to the KD model, there were no differences in inhibitory synapse number and the frequency of mIPSCs between genotypes; in fact we observed an increase in the % co-localization between GAD67 and gephyrin, suggesting increased expression of GAD-67 as a likely compensatory mechanism for reduced glutamatergic inputs. Synaptic homeostasis mechanisms have been thoroughly demonstrated to maintain neuronal excitability (Davis and Bezprozvanny, 2001; Turrigiano, 2012) and GAD67 is a target for activity-dependent regulation (Lau and Murthy, 2012); however changes in expression of gephyrin has not been ruled out.

The inability to compensate the excitatory/inhibitory imbalance in KO neurons is likely due to the combined altered expression of synapsin I and α_1A_ and α_1H_ Ca_V_ channels. Synapsin phosphoproteins play an important role in the regulation of axonogenesis, synaptogenesis and the modulation of neurotransmitter release (Hilfiker et al., 1999; Ferreira and Rapoport, 2002; Evergren et al., 2007; Bykhovskaia, 2011). In parallel, pre- and post-synaptic Ca_V_ channel subunits are directly involved in the formation and maintenance of synapses by interacting with synapse organizers (Brodbeck et al., 2002; Nishimune et al., 2004; Eroglu et al., 2009; Kurshan et al., 2009; Nishimune, 2011). We studied the status of somatic calcium currents only, but western blot results are indicative of global Ca_V_ protein levels in pre- and post-synaptic sites altogether. P/Q-type channels are preferentially located in presynaptic terminals throughout the brain; they have a prominent role in controlling neurotransmitter release that increases during development (Nudler et al., 2003; Inchauspe et al., 2004; Giugovaz-Tropper et al., 2011); thus decreased presynaptic α_1A_ could alter the number of excitatory synapses in the KLHL1 KO. Q and N-type calcium channels mediate synaptic transmission in the hippocampus (Scholz and Miller, 1995; Wheeler et al., 1995; Pravettoni et al., 2000), and P/Q-, N- and R-type channel openings account for ~50% of all spontaneous glutamate release at rat cultured hippocampal synapses (Scholz and Miller, 1995; Ermolyuk et al., 2013); on the other hand, N- and L-type but not P/Q-type channel activity generate the spontaneous release of GABA in hippocampal granule neurons (Goswami et al., 2012). Therefore decreased P/Q channel levels and activity could affect excitatory neurons to a greater extent than inhibitory neurons. Furthermore, regulation of spontaneous synaptic release of glutamate by presynaptic Ca_V_3.2 channels has been reported for dorsal horn neurons, raising the possibility that alterations in T-type current activity could also have a deleterious role in pre-synaptic function of excitatory hippocampal neurons (Bao et al., 1998; Jacus et al., 2012; Rozanski et al., 2013; Fekete et al., 2014). In contrast, L-type channels are preferentially located at postsynaptic sites and have less effect on neurotransmitter release (Kamiya et al., 1988; Pravettoni et al., 2000), therefore up-regulation of L-type currents unlikely compensated for deficits in presynaptic calcium currents in the KO. Finally, altered astrocyte function due to the absence of KLHL1 could be possible and has not been ruled out as a factor contributing to the synaptic deficits observed in the KLHL1 KO (Ullian et al., 2001; Christopherson et al., 2005; Jiang et al., 2007; Südhof, 2012).

The KLHL1 KO mouse model highlights the importance of KLHL1 in the modulation of Ca_V_2.1 and Ca_V_3.2-mediated calcium currents, and revealed compensatory mechanisms to maintain calcium homeostasis. Overall, ablation of KLHL1 leads to an initial decrease in calcium current in cultured neurons, which was compensated for by homeostatic up-regulation of voltage dependent calcium currents not subjected to modulation by KLHL1, such as L-, N- and α_1G_ T-type currents. However, despite the up-regulation of somatic HVA and LVA current densities, possible alterations in calcium channel composition at presynaptic sites and down-regulation of synapsin I resulted in decreased number of excitatory synapses and altered balance between excitatory and inhibitory inputs onto hippocampal neurons, corroborating a synaptic function for KLHL1 (Aromolaran et al., 2007, 2009, 2010; Perissinotti et al., 2014). These deficits may become significant during process of sub-optimal function, such as aging or in disease.

## Author’s contributions

Erika S. Piedras-Rentería conception of research; Michael D. Koob’s laboratory generated the KLHL1-knockout mice; Erika S. Piedras-Rentería and Paula P. Perissinotti designed the experiments; Paula P. Perissinotti, Elizabeth A. Ethington, Erik Almazan, Elizabeth Martínez-Hernández, Jennifer Kalil and Erika S. Piedras-Rentería performed experiments; Erika S. Piedras-Rentería supervised the experiments; Paula P. Perissinotti and Erika S. Piedras-Rentería analyzed data, interpreted results of experiments; Paula P. Perissinotti and Erika S. Piedras-Rentería prepared figures; Erika S. Piedras-Rentería and Paula P. Perissinotti wrote the manuscript; Paula P. Perissinotti and Erika S. Piedras-Rentería edited and revised the manuscript. Erika S. Piedras-Rentería approved the final version of the manuscript.

## Conflict of interest statement

The authors declare that the research was conducted in the absence of any commercial or financial relationships that could be construed as a potential conflict of interest.
